# Differences Among Racial and Ethnic Minority Groups in the Unmet Existential and Supportive Care Needs of People Receiving Dialysis

**DOI:** 10.1001/jamainternmed.2022.1677

**Published:** 2022-07-11

**Authors:** Catherine R. Butler, Rashmi K. Sharma, Nwamaka D. Eneanya, Gwen M. Bernacki, Jasleen K. Ghuman, J. Randall Curtis, Ann M. O’Hare

**Affiliations:** 1 Kidney Research Institute and Department of Medicine, Division of Nephrology, University of Washington, Seattle; 2 Veterans Affairs Health Services Research & Development Seattle-Denver Center of Innovation for Veteran-Centered and Value-Driven Care, Seattle, Washington; 3 Department of Medicine, Division of General Internal Medicine, University of Washington, Seattle; 4 Cambia Palliative Care Center of Excellence, University of Washington, Seattle; 5 Palliative and Advanced Illness Research Center, Perelman School of Medicine, University of Pennsylvania, Philadelphia; 6 Department of Medicine, Division of Cardiology, University of Washington and Veterans Administration Puget Sound Geriatric Research Education and Clinical Center, Seattle; 7 Department of Medicine, Division of Pulmonary, Critical Care and Sleep Medicine, University of Washington, Seattle

## Abstract

This cohort study examines differences regarding existential and supportive care needs for patients with kidney disease between individuals of racial and ethnic minority groups compared with White individuals.

Racial and ethnic minority groups experience a disproportionate burden of kidney disease in the US, and are less likely than White individuals to receive recommended kidney care.^1,2^ Advanced kidney disease and treatments, such as dialysis and transplant, can be life changing, physically demanding, and psychosocially challenging, and many patients report unmet existential and supportive care needs.^3^ To our knowledge, whether these types of care needs differ between racial and ethnic minority groups is not known.

## Methods

We administered a survey about palliative and end-of-life care to 997 English-speaking adults who were receiving maintenance dialysis at 31 nonprofit dialysis facilities in Seattle, Washington, and Nashville, Tennessee, between April 2015 and October 2018 (eFigure and eMethods in the Supplement).^4^ This study was approved by the institutional review board at the University of Washington in Seattle, Washington, and all study participants provided written informed consent. We report analyses conducted among the 948 study participants who reported their race as Asian American or Native Hawaiian or other Pacific Islander, as Black or African American, or as White. Because of the small number of participants who self-identified as Hispanic, we were unable to stratify exposure groups by this ethnicity. Unmet existential and supportive care needs were ascertained using an adaptation of an existing instrument.^3^

We examined the association between racial and ethnic group and each unmet care need using a logistic regression that was adjusted for age group, sex, Hispanic ethnicity, years receiving dialysis, recruitment site, self-reported health status, educational attainment, and the importance of religious or spiritual beliefs. We described overall between-group differences using *P* values for likelihood ratios. We presented marginal proportions and risk differences for Asian American or Native Hawaiian or other Pacific Islander participants and Black participants compared with White participants, which were averaged across the distribution of covariates in the study sample. We considered *P* values of <.05 to be statistically significant. Analyses were performed using Stata (version 16.0; StataCorp).

## Results

The analytic cohort was comprised of 113 Asian American or Native Hawaiian or other Pacific Islander (12%), 270 Black (29%), and 565 White participants (60%) (Table). In adjusted analyses (Figure), Asian American or Native Hawaiian or other Pacific Islander participants and Black participants were significantly more likely than White participants to report wanting to learn about treating pain (adjusted proportions of 55% for Asian American or Native Hawaiian or other Pacific Islander participants and 53% for Black participants vs 39% for White participants) and treating symptoms of kidney disease (70% and 68% vs 52%, respectively). These groups were also significantly more likely to report wanting someone to talk with about the meaning of life (23% and 17% vs 8%) and finding peace of mind (31% and 24% vs 17%), wanting help in sharing thoughts and feelings with those close to them (41% and 32% vs 22%), and finding spiritual resources (27% and 19% vs 11%).

**Table.** Participant Characteristics

Characteristic	No. (%)		
	Asian American or Native Hawaiian or other Pacific Islander (n = 113)	Black (n = 270)	White (n = 565)
Age, mean (SD), y	60 (14)	59 (13)	65 (14)
Sex			
Female	42 (37)	128 (47)	248 (44)
Male	71 (63)	142 (53)	317 (56)
Ethnicity			
Non-Hispanic	108 (96)	253 (94)	522 (92)
Hispanic	4 (4)	4 (2)	42 (7)
Missing	1 (1)	13 (5)	1 (0.2)
Recruitment site			
Seattle, Washington	112 (99)	145 (54)	442 (78)
Nashville, Tennessee	1 (1)	125 (46)	123 (22)
Type of dialysis			
Peritoneal dialysis	0	1 (0.4)	4 (1)
Hemodialysis	112 (99)	263 (97)	555 (98)
Missing	1 (1)	6 (2)	6 (1)
Years receiving dialysis			
≤1	34 (30)	61 (23)	169 (30)
>1 to 5	55 (49)	126 (47)	275 (49)
>5	24 (21)	83 (31)	120 (21)
Missing	0	0	1 (0.2)
Self-rated health status			
Very good or excellent	26 (23)	47 (17)	113 (20)
Good	48 (43)	105 (39)	210 (37)
Fair or poor	39 (35)	118 (44)	242 (43)
Highest educational level			
Less than high school	8 (7)	44 (17)	64 (11)
High school	32 (29)	99 (37)	185 (33)
Some college	27 (24)	44 (17)	91 (16)
Graduated from college or post graduate training	45 (40)	80 (30)	223 (40)
Missing	1 (1)	3 (1)	2 (0.4)
Response to statement about importance of religious and spiritual beliefs			
Definitely true	57 (50)	170 (63)	218 (39)
Tends to be true	25 (22)	57 (21)	143 (25)
Tends not to be true	17 (15)	14 (5)	103 (18)
Definitely not true	12 (11)	25 (9)	97 (17)
Missing	2 (2)	4 (2)	4 (1)

**Figure.**
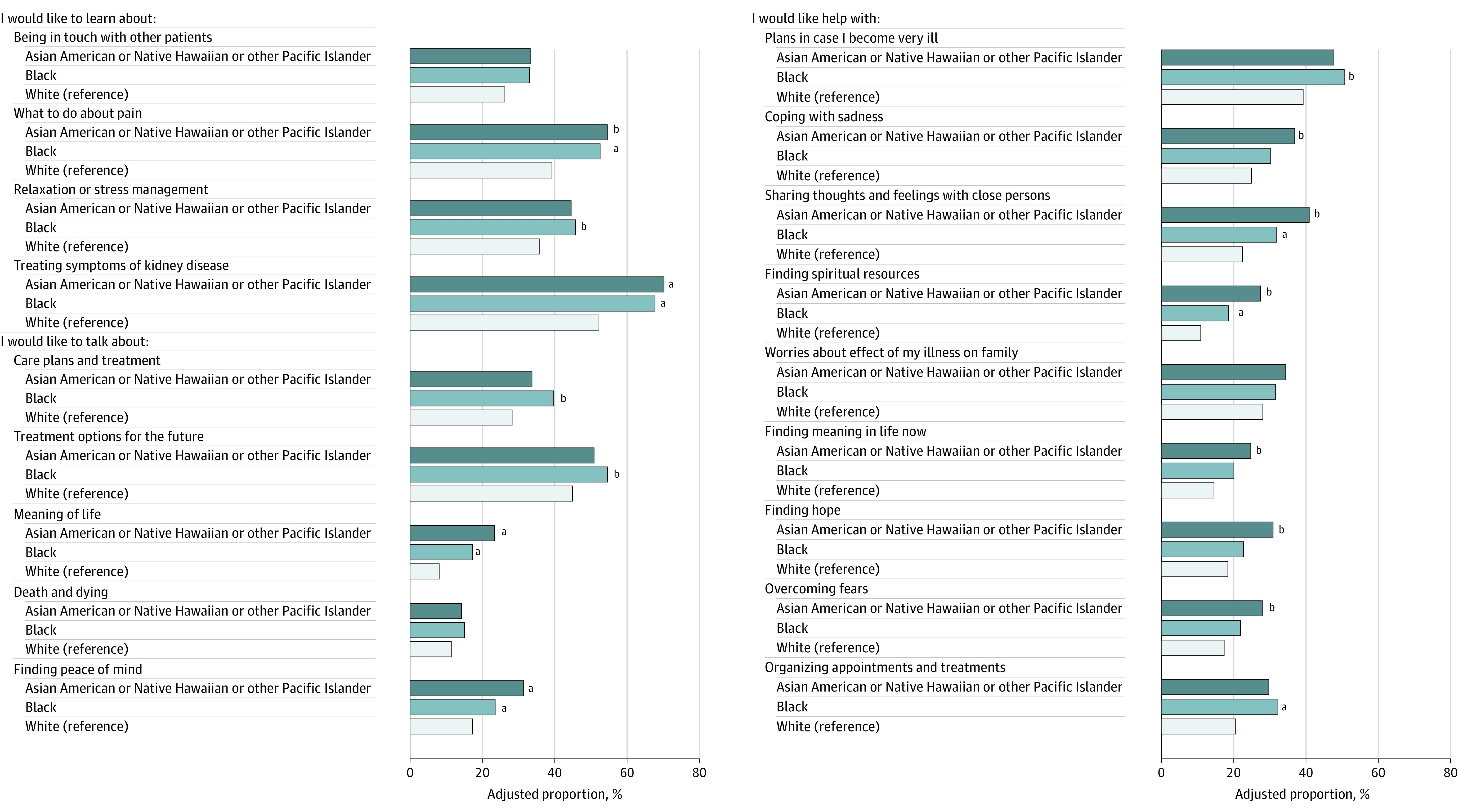
Existential and Supportive Care Needs Among Racial and Ethnic Groups of People Who Were Receiving Dialysis Adjusted proportions adjusted for age group, sex, Hispanic ethnicity, recruitment site, years receiving dialysis category, self-rated health status, educational attainment, and reported importance of spirituality. ^a^*P* < .003 (Bonferroni adjustment for multiple comparisons). ^b^*P* < .05.

Asian American or Native Hawaiian or other Pacific Islander participants, but not Black participants, were more likely than White participants to report wanting help in coping with sadness, finding meaning in life, finding hope, and overcoming fears. Black participants, but not Asian American or Native Hawaiian or other Pacific Islander participants, were significantly more likely than White participants to report wanting to learn more about relaxation or stress management and that they would like to have someone to talk to about their care plan and treatment options for the future. They were also more likely to indicate that they would like help with making plans in case they were to develop severe illness and with organizing appointments and treatments.

## Discussion

In this survey study of people who were receiving maintenance dialysis in 2 US metropolitan areas between April 2015 and October 2018, we found that those who identified as Asian American or Native Hawaiian or other Pacific Islander or as Black were more likely than White participants to report a wide range of unmet existential and supportive care needs. These findings contribute to a body of work illuminating disparities in care across a range of health care settings and indicate a need to better understand the lived experiences of those who identify as Asian American or Native Hawaiian or other Pacific Islander or as Black in receiving dialysis and the role of systemic racism in shaping their care. While we were able to capture several measures of social environment, this study includes limited information about social determinants of health or more precise measures of the experience of racism. Future work should also include more granular racial and ethnic categories and a greater representation of Latinx people. The study results suggest a need for practice interventions and policy initiatives to promote equitable access to health services, including palliative and supportive care.^5^ This work should be undertaken in partnership with stakeholders from diverse racial, ethnic, and socioeconomic backgrounds.^6^

## Notes

Supplement. eFigure. Cohort derivation eMethods.

## References

[1] United States Renal Data System. 2020 USRDS Annual Data Report: Epidemiology of kidney disease in the United States. National Institute of Diabetes and Digestive and Kidney Diseases; 2020.

[2] Smedley BD, Stith AY, Nelson AR, eds. Unequal Treatment: Confronting Racial and Ethnic Disparities in Health Care. National Academies Press; 2003. 25032386

[3] Davison SN, Jhangri GS. Existential and supportive care needs among patients with chronic kidney disease. J Pain Symptom Manage. 2010;40(6):838-843. doi:10.1016/j.jpainsymman.2010.03.015 20739142

[4] O’Hare AM, Kurella Tamura M, Lavallee DC, . Assessment of self-reported prognostic expectations of people undergoing dialysis: United States Renal Data System Study of Treatment Preferences (USTATE). JAMA Intern Med. 2019;179(10):1325-1333. doi:10.1001/jamainternmed.2019.2879 31282920 PMC6618699

[5] Bailey ZD, Krieger N, Agénor M, Graves J, Linos N, Bassett MT. Structural racism and health inequities in the USA: evidence and interventions. Lancet. 2017;389(10077):1453-1463. doi:10.1016/S0140-6736(17)30569-X 28402827

[6] Cooper LA, Hill MN, Powe NR. Designing and evaluating interventions to eliminate racial and ethnic disparities in health care. J Gen Intern Med. 2002;17(6):477-486. doi:10.1046/j.1525-1497.2002.10633.x 12133164 PMC1495065

