# Supplementary material for: Differences Among Racial and Ethnic Minority Groups in the Unmet Existential and Supportive Care Needs of People Receiving Dialysis
Source: JAMA Intern Med. 2022 Jul 11;182(9):992–5. doi: 10.1001/jamainternmed.2022.1677 (PMC9274444; doi:10.1001/jamainternmed.2022.1677)
Supplement: Supplement. — eFigure. Cohort derivation eMethods. [file jamainternmed-e221677-s001.pdf]

Supplemental Online Content

Butler CR, Sharma RK, Eneanya ND, et al. Differences among racial and ethnic minority groups in the unmet existential and supportive care needs of people receiving dialysis. *JAMA Intern Med*. Published online July 11, 2022. doi:10.1001/jamainternmed.2022.1677

eFigure. Cohort derivation
eMethods.

This supplemental material has been provided by the authors to give readers additional information about their work.

© 2022 Butler CR et al. *JAMA Internal Medicine.*

eFigure. Cohort derivation

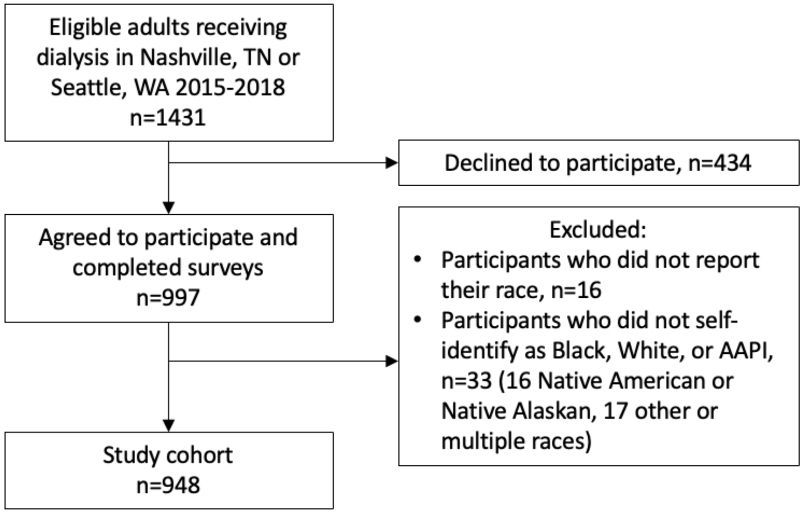

Abbreviations: Asian or Native Hawaiian or other Pacific Islander, AAPI.

© 2022 Butler CR et al. *JAMA Internal Medicine.*

eMethods

We administered a survey designed to elicit the palliative, and end-of-life care needs of patients with kidney disease to individuals receiving maintenance dialysis at 31 nonprofit dialysis facilities in Seattle, WA and Nashville, TN between April 2015 and October 2018. Eligible participants were aged 21 years or older, fluent in English, and cognitively able to provide written informed consent. Study staff approached 1431 eligible patients receiving maintenance dialysis at participating facilities at the time of survey administration and recruited a pragmatic sample of 997 participants (69.7% participation rate) (**eFigure 1**). Study participants were given the option to complete the survey while receiving dialysis (by either completing the form themselves or having a study coordinator record their verbal responses to survey questions) or return the survey at a later time. The analyses described here were conducted among the subset of participants who reported their race as Black or African American, Asian American or Native Hawaiian or other Pacific Islander (AAPI), or White. We excluded 16 participants who did not report their race and 16 participants who identified as Native American or Native Alaskan and 17 who identified as other or multiple races because there were too few participants in each of these groups to support estimation, yielding an analytic cohort of 948 participants (66.2% of those originally invited to participate). Due to the small number of participants who self-identified as Hispanic ethnicity, we were unable to stratify exposure groups by ethnicity.

Supportive and existential care needs.

Using an adaptation of an existing survey instrument from Davison et al., participants were asked about: 1) whether they would like to learn more about: how to be in touch with other patients with kidney disease, what they could do about pain, relaxation or stress management, and treating the symptoms of kidney disease; 2) whether they would like help with: making plans in case they were to become very ill, learning to cope with feelings of sadness, sharing thoughts and feelings with those close to them, finding spiritual resources, worries about the effect of their illness on family, finding meaning in life now, finding hope, overcoming fears, and organizing their appointments and treatments; and 3) whether they would like to talk about: their care plan and treatments, treatment options for the future, the meaning of life, dying and death, and finding peace of mind.

© 2022 Butler CR et al. *JAMA Internal Medicine.*

Demographic characteristics.

In addition to self-reported race, the survey included questions about age, sex, Hispanic ethnicity, duration of dialysis treatment, self-rated health status (excellent, very good, good, fair, or poor), and highest educational level (less than high school, graduated from high school, some college or trade school, graduated from college or trade school, or post-graduate training). To ascertain the importance of religious or spiritual beliefs, participants were also asked to respond to the statement “my religious or spiritual beliefs are what really lie behind my whole approach to life” (definitely true, tends to be true, tends not to be true, or definitely not true).

Statistical analysis.

We used proportions and mean values (with standard deviations) to describe categorical and continuous variables, respectively. We used chi-square tests to describe unadjusted differences in responses to survey questions between racial groups. White participants served as the reference group for adjusted statistical analyses they were the largest group. Multivariate analyses were conducted using logistic regression adjusted for age group (21-59 years, 60-74, ≥75 years), sex, ethnicity, years on dialysis (≤1 year, >1year-5 years, >5 years), recruitment site, self-reported health status (categorized as very good or excellent, good, and fair or poor), educational status, and spiritual beliefs. Participants missing information on outcomes were excluded from the relevant analyses. Overall between-group differences in survey responses are described using p-values for likelihood ratios. Comparisons between responses of Black and of AAPI participants with the reference group of White participants are presented as adjusted proportions and risk differences (with 95% confidence intervals) using logistic regression models and the Stata margins function. Because our goal was to conduct descriptive analyses, p-values of <0.05 were considered statistically significant. However, we also denote p-values that met the more conservative threshold for statistical significance defined by Bonferroni correction for multiple comparisons ($p<0.003$).

This study was approved by the institutional review board at the University of Washington in Seattle, WA and all study participants provided written informed consent.

© 2022 Butler CR et al. *JAMA Internal Medicine.*
